# In vitro characterization of immune modulating drug-eluting immunobeads towards transarterial embolization in cancer

**DOI:** 10.1038/s41598-022-26094-1

**Published:** 2022-12-19

**Authors:** Ayele H. Negussie, Andrew S. Mikhail, Joshua W. Owen, Natalie Hong, Camella J. Carlson, Yiqing Tang, Kendal Paige Carrow, Michal Mauda-Havakuk, Andrew L. Lewis, John W. Karanian, William F. Pritchard, Bradford J. Wood

**Affiliations:** 1 Center for Interventional Oncology, Radiology and Imaging Sciences, NIH Clinical Center, National Institutes of Health, Bethesda, MD USA; 2 Biocompatibles UK Ltd (a BTG International Group Company), Lakeview, Riverside Way, Watchmoor Park, Camberley, GU15 3YL Surrey UK; 3 National Cancer Institute, National Institutes of Health, Bethesda, MD USA

**Keywords:** Cancer, Immunology, Chemistry

## Abstract

Hepatocellular carcinoma (HCC) is an aggressive liver cancer with limited effective treatment options. In this study, we selected TLR agonists imiquimod (IMQ), gardiquimod (GARD), GS-9620 and DSR 6434, and a small molecule checkpoint inhibitor, BMS-202, for characterization of drug loading and release from radiopaque embolic beads (DC Bead LUMI) for potential use in image-guided transarterial embolization (TACE) of HCC. The maximum drug loading capacity and amount of drug released over time were determined by high performance liquid chromatography and compared with the commonly used anthracycline, doxorubicin hydrochloride (Dox). Maximum drug loading was 204.54 ± 3.87, 65.97 ± 1.54, 65.95 ± 6.96, 65.28 ± 3.09, and 148.05 ± 2.24 mg of drug per milliliter of DC Bead LUMI for Dox, GARD, DSR 6434, IMQ, and BMS-202, respectively. Fast loading and subsequent rapid release in saline were observed for IMQ, GARD, and DSR 6434. These drugs could also be partially removed from the beads by repeated washing with de-ionized water suggesting weak interaction with the beads. Aggregation of IMQ was observed in water and saline. GS-9620 partially decomposed in the solubilizing solution, so loading and release were not characterized. Compared to TLR agonists, slower loading and release were observed for Dox and BMS-202. Potential factors influencing drug loading into and release from DC Bead LUMI including steric hinderance, hydrophobicity, drug pKa, and the electrostatic nature of the beads are discussed. The maximum loading capacity of BMS-202 and Dox in DC Bead LUMI exceeded the maximum theoretical loading capacity of the beads expected from ionic interaction alone suggesting additional drug-bead or drug-drug interactions may play a role. Slightly more release was observed for BMS-202 at early time points followed by a slower release compared to Dox. Further study of these drug-bead combinations is warranted in search of new tools for locoregional delivery of immune-modulating agents for treatment of HCC via drug-eluting bead chemoembolization.

## Introduction

Hepatocellular carcinoma (HCC) is an aggressive liver cancer with poor prognosis even following potentially curative treatment options^1,2^. Expression of Toll-like receptors (TLRs) and immune checkpoints has immunomodulatory implications and may serve as targets for pharmacotherapy. For instance, activated TLRs are involved in induction of antigen-specific immunity as well as inflammatory responses. Immune checkpoints on tumor cells are implicated in immune escape and self-tolerance, and their inhibition has instigated a major paradigm shift in oncology including in the treatment of advanced HCC.

Clinical trials have demonstrated benefits of blockade of immune inhibitory pathways leading to U.S. Food and Drug Administration approval of anti-PD-1 checkpoint inhibitors (CPI) for the treatment of advanced HCC^3–7^. The use of low molecular weight CPI, instead of monoclonal antibodies, could improve tumor penetration and facilitate incorporation into drug delivery systems such as embolic beads for targeted locoregional delivery^8^. Understanding disease pathogenesis, tumor microenvironment and identification of clinically relevant molecular phenotypes of HCC^9,10^ may help support the premise of immunotherapy with TLR agonists and/or small molecule CPI as an attractive therapeutic approach.

HCC develops in the liver, where various immune cells reside and play a role in promoting or inhibiting tumor growth^11–18^. TLRs function primarily as pattern recognition receptors capable of initiating immune responses upon exposure to microbial components or other natural or synthetic agonists. TLRs are also expressed by tumor cells including HCC and their activation plays an important role in tumor proliferation, resistance to apoptosis, and invasion (e.g., TLR 2 and TLR 4)^19,20^. In addition to potential involvement in tumor-promoting signals, TLRs may also contribute to anti-tumor immune responses^19–21^. Moreover, TLRs bind to small molecules (agonists) generating an immune response and may potentially serve as novel therapeutic targets for HCC therapy^12,22–29^. A number of synthetic TLR agonists including gardiquimod (GARD), DSR 6434, and GS-9620 have been evaluated in pre-clinical^27,30–32^ studies and imiquimod (IMQ) in clinical studies^33–35^ for various cancer treatments. In addition, small molecule PD-1/PD-L1 CPI, such as BMS-202 and analogues, have been reported to show potent PD-1/PD-L1 binding inhibition^36,37^. However, systemic administration of these drugs may be limited by their modest aqueous solubility and systemic toxicity. Therefore, loading into embolic beads for locoregional treatment of HCC is an alternative delivery strategy^38^.

We have previously demonstrated the in vitro proliferative effects of a panel of immunomodulators on peripheral blood mononuclear cells of woodchucks and release of DSR 6434 and BMS-202 from embolic beads^8^. The in vivo biocompatibility and safety of imageable beads loaded with doxorubicin (Dox) and irinotecan have also been reported^39,40^. In this study, immune-modulating drugs were loaded into radiopaque embolic beads by salt formation and drug elution from the beads was characterized and compared to the reference drug Dox. Among candidates considered, TLR agonists IMQ, GARD, GS-9620, and DSR 6434 and small molecule CPI, BMS-202, were chosen for this study based on their potential to be converted into cationic forms during the process of aqueous solubilization which is critical for loading into embolic beads via ionic interaction. In addition, solubility factors and proven efficacy in vivo were also major considerations. DC Bead LUMI is an established radiopaque embolic bead that has been loaded with chemotherapeutic agents such as Dox, irinotecan, and other drugs for image-guided TACE of liver malignancies in preclinical and clinical studies^41–45^. Factors influencing maximum drug loading and elution kinetics from DC Bead LUMI in relation to LC Bead are presented for Dox and BMS-202.

## Material and methods

### Materials

Embolic beads DC Bead LUMI (70–150 µm in diameter) and LC Bead (70–150 µm) were obtained from Biocompatibles, UK Ltd., [formerly a BTG plc, now Boston Scientific Inc. Company], Farnham, UK). The base forms of GARD (≥ 98%, Enzo, Farmingdale, NY, ≥ 98%), IMQ (TCI, Nihonbashi-honcho, Chuo-ku, Tokyo > 98.0%), GS-9620 (Advanced ChemBlocks Inc., Burlingame, CA), DSR 6434 (R&D Systems, Minneapolis, MN), BMS-202 (Abcam, Cambridge, MA) and Dox (LC Laboratories, Woburn, MA) were used.

### Elemental analysis

DC Bead LUMI was analyzed for its elemental composition by MEDAC Inc. (Egham, Surrey, UK); and the sulfur content was used to estimate the drug loading capacity of the bead, as this provides a measure of the drug-binding sulfonate residues^46^.

### pKa measurements for GARD and DSR 6434

pKa measurements were performed by Pion Inc, UK Ltd using known methods as previously reported^47–50^ or calculated by using software MoKa (Molecular Discovery, Borehamwood, Hertfordshire, UK, https://www.moldiscovery.com/software/moka/#references) or directly obtained from the literature. For GARD and DSR 6434, a triple titration was carried out under methanol–water co-solvent conditions from pH 2.0–12.0 at concentrations of 2.3–1.3 mM (the methanol mixing ratio varied from 49.0 to 28.9% w/w) and 1.5–0.9 mM (the methanol mixing ratio varied from 49.6 to 29.1% w/w), respectively. No precipitation of the sample from solution was observed for both drugs.

### Drug solubilization

The base form of each drug was suspended in deionized water and titrated with 0.1 N hydrochloric acid (Fisher Scientific, Hampton, NH) while stirring until all the base was solubilized and the volume adjusted to a final concentration of 2.5 mg/mL. The resulting solution was frozen, then lyophilized to yield solid drug-hydrochloride salt or used in its solubilized form for loading into DC Bead LUMI. BMS-202 did not solubilize using 0.1 N HCl and was instead solubilized using an equimolar amount of methanesulfonic acid. Doxorubicin.HCl was readily solubilized and used without modification. HPLC analysis for all drugs was performed with an acetonitrile (0.1% trifluoroacetic acid) and deionized water (0.1% trifluoroacetic acid) mobile phase (Dox: [36:64], IMQ: [36:64], GARD: [40:60], DSR 6434 [30:70], BMS-202 [50:50], and GS-9620 [20:80]) at a flow rate of 1 mL/min. Drug concentrations in loading and release media were evaluated by using a ZORBAX Eclipse Plus C18 reversed-phase column (Agilent, Santa Clara, CA), the solvent system indicated above, and a UV detector at a wavelength of 242 nm for IMQ, 230 nm for GARD, and 240 nm for DSR 6434 and BMS-202. Fluorescent detection at $\lambda$_abs/emis_ 480/530 nm was used for Dox. A ZORBAX Eclipse Plus C8 reversed-phase column (Agilent) and UV detector at wavelength of 230 nm were used for GS-9620 analysis. The decomposition product of GS-9620 was analyzed with reversed phase high-performance liquid chromatography (Rp-HPLC) and LC/MS.

### Drug loading studies

Drug loading was performed in triplicate as previously reported^45^ with slight modifications. Briefly, 1.3 molar equivalent of each drug was mixed with one molar equivalent of sulfonate group of DC Bead LUMI or LC Bead. The drug-bead mixture was agitated with upright rocking motion at a set maximum rocking speed of 10 which corresponds to 70 rpm, for 144 h at ambient temperature (Ward's^®^ Rotating Mixers, VWR, Bridgeport, NJ). At specified time points (5, 15, 30 and 45 min and then hourly for eight hours followed by 24, 48, 72 and 144 h) a 10 µL aliquot was withdrawn from the loading solution to quantify drug loading based on the change in concentration over time. Washing of beads is often a critical step in drug release studies as any drug left unbound to the beads affects the release profile and exaggerates the early burst release. Therefore, drug-loaded beads were washed with deionized water until no more drug residue was observed with HPLC for BMS-202 and Dox and stored until further analysis. However, washing was discontinued for TLR drugs as drug was continually observed even after multiple washes. The concentration of drug in each aliquot of loading solution at each time point was quantified by HPLC as described above using a linear standard curve obtained from solutions of known concentration of each drug (7.8–125 μg/mL) *versus* the detector response as area under the curve. The amount of drug loaded at the specified time points was calculated by subtracting the amount of drug remaining from the initial amount of drug in the loading solution and the cumulative amount of drug withdrawn for sampling. The maximum drug loaded in the beads was calculated once no further change in the concentration in the loading solution could be measured and before washing the beads. The relative percent drug loaded was defined as the maximum amount of drug loaded as a percent of the amount of SO_3_^−^ ions available in the beads on a molar basis using the following formula and the results are presented as the mean ± SD.

$$Relative\; \% \;Drug\;load = 100 \times \frac{maximum\;amount\;of\;drug\;loaded}{{Theoretical\;loadable\;drug\;amount}}$$

### Size and appearance of drug-loaded beads

The appearance of DC bead LUMI before and after loading with Dox and IMQ and after drug elution, as well as LC Bead before and after loading with Dox and after Dox elution were examined by use of an upright microscope (Zeiss Axioimager M1, Thornwood, NY) equipped with a color CCD camera (AxioVision, Zeiss) at 10${\times}$ magnification as previously reported^40^.

### Drug release from beads

Drug release experiments were performed in triplicate. Briefly, drug-loaded DC Bead LUMI or LC Bead (62.5 µL) were placed in vessels with 50 mL of saline (0.9 mg/mL, pH 7.4) and were agitated with upright rocking motion at a set maximum rocking speed of 10 (70 rpm) for 144 h at 37 °C using Ward's^®^ Rotating Mixer. At scheduled time intervals identical to the loading time points, 1 mL of release media was withdrawn and used for drug quantification. To maintain 50 mL total elution volume, 1 mL of fresh saline pre heated to 37 °C was replaced at each timepoint. The concentration of drug in each aliquot was quantified by HPLC using regression curve constructed from known concentration of each drug (linear range 0.98–62.5 µg/mL) as described in Sect. 2.4. The cumulative amount of drug released at each time point was calculated by multiplying the concentration of each sample aliquot by the total volume (50 mL) of release media and adding the sum of the amount of drug removed for sampling at prior time points. The results are presented as the mean ± SD of the molar amount of drug released and as a percent of the initial amount of drug loaded, over time. The 50 mL volume was chosen to ensure infinite sink conditions where the maximum possible concentration of eluted drug would be at least 10 times more dilute than the maximum solubility. This ensures the rate of release is not affected by solution concentration but is only a function of interactions of the bead, drug, and saline.

## Results and discussion

### Elemental composition of DC Bead LUMI

The elemental composition of a typical dry DC Bead LUMI was 30.7% C, 3.44% H, 1.33% N, 2.85% S, 44.6% I and 17.13% O and Na combined. The 2.85% S corresponds to 2.87 $\times$ 10^–4^ mol/mL of sulfonate group indicating a maximum loading of 0.287 mmol of drug in its salt form (hydrochloride, sulfonate, etc.) in 1 mL of the beads assuming a 1:1 drug: sulfonate binding ratio. The binding ratio may change if there are additional protonatable moieties within the drug structure, such as mitoxantrone^51^ or vandetanib^45^.

### pKa of the drugs

**Figure 1:**
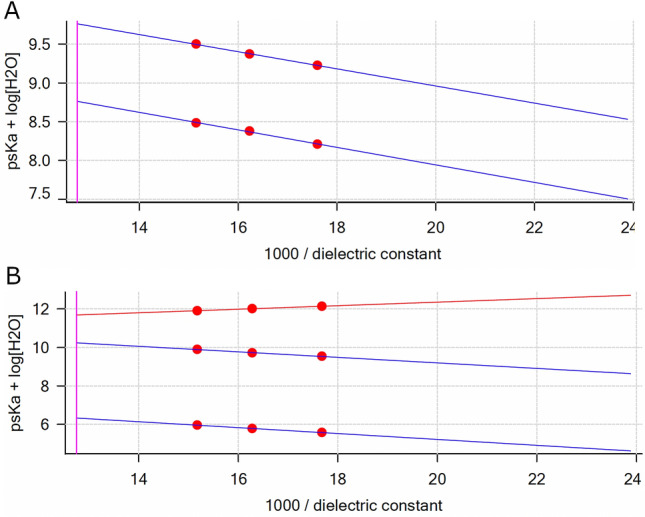
Yasuda-Shedlovsky plots for GARD (**A**) and DSR 6436 (**B**) in their protonated forms: GARDH (R^2^ = 0.9983), GARDH_2_ (R^2^ = 0.9967) and DSR 6434H (R^2^ = 0.9959), DSR 6434H_2_ (R^2^ = 0.9986), DSR 6434H_3_ (R^2^ = 1.000). Values for x = 100/έ in 29, 39 and 50 wt % MeOH in water. The x-intercepts at 100/έ = 1.30 are the psKa + log [H_2_O] values at zero MeOH content (aqueous).

For GARD, two pKas, with aqueous values of 7.02 ± 0.02 and 8.02 ± 0.02, and for DSR 6434, three pKas, with aqueous values of 4.58 ± 0.01, 8.50 ± 0.02 and 9.94 ± 0.02, were determined from the data collected by Yasuda-Shedlovsky extrapolation of the individual results obtained (Table 1 and Fig. 1). pKas for IMQ, BMS-202, and Dox are reported from literature^52–54^ while pKas for GS-9620 were calculated by using software MoKa.

**Table 1:** pKa of TLR agonists and inhibitor of PD-1/PD-L1; and pH at which each drug dissolved for loading.

Drug	GARD	IMQ	GS-9620	DSR 6436	BMS-202	Dox
pKa	7.02 & 8.02 (measured) 6.97 and 7.71 (calculated)	7.3 (literature) ^52^	6.53, 8.88, 9.50 (calculated)	4.58, 8.50 & 9.94 (measured)	7.74 (literature) ^54^	8.2 (literature) ^53^
pH (T in °C)	–	2.06 (20.1)	–	1.65 (20.1)	2.11 (20.1)	4.2 (21.1)

### Drug solubilization and loading into DC Bead LUMI

Salt formation is a preferred technique to solubilize drug in water. Using this approach, all drugs except BMS-202 were solubilized into their hydrochloride salt forms. BMS-202 did not solubilize upon addition of 0.1 N HCl and instead was converted into a mesylate salt by solubilizing with methanesulfonic acid. GS-9620 was not stable in the chosen solubilizing conditions as two peaks were observed on analysis with HPLC. The individual peaks were further analyzed by liquid chromatography-mass spectrometry and one of the peaks was found to be the parent drug with m/z = 410 atomic mass units (amu) while the second peak was two mass units less than the parent peak, m/z = 408 amu. Further analysis was not conducted to fully characterize the unknown sample, as it was beyond the scope of this work.

**Figure 2:**
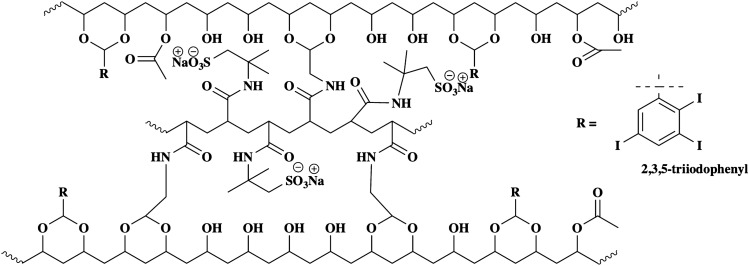
Chemical structure of DC Bead LUMI.

The introduction of a radiopacifier on DC Bead LUMI changes its properties compared to LC Bead in the following ways. First, DC Bead LUMI is denser because of the attached three-iodine-molecule-containing moiety within the polymer matrix which results in shrinkage of the beads. As a result, DC Bead LUMI has a large population of sulfonate groups responsible for drug loading compared to LC Bead of the same volume^42^. However, the resulting radiopaque beads may have narrower pore sizes limiting percolation of the drug solution. Second, DC Bead LUMI is hydrophobic because of the attached phenyl groups resulting in hydrophobic interaction with drugs having hydrophobic properties. Finally, the radiopacifiers, three molecules of iodine attached to the phenyl group, are relatively large molecules so that they could sterically hinder incoming bulky drug molecules from freely interacting with the sulfonate groups (Fig. 2).

Theoretical and previous experimental evidence showed that for every equivalent of sulfonate group in the LC Bead, one equivalent of Dox or irinotecan is ionically bound^46,55^. Other drugs, such as mitoxantrone, can interact with the beads at multiple binding sites due to the presence of two protonated amines^51^. In one milliliter of LC Bead, 7.7 $\times$ 10^–5^ mol of negatively charged sulfonate groups are available for carrying protonated drug^46^. Loading capacity for every milliliter of DC Bead LUMI, according to the calculated sulfonate groups from the elemental analysis data, was 2.87 $\times$ 10^–4^ mol indicating DC Bead LUMI can load up to 3.7-fold more drug on a molar basis than LC Bead. As a result, 166.50 mg of Dox, 114.94 mg of DSR 6434, 89.95 mg of GARD, 68.97 mg of IMQ, and 120.40 mg of BMS-202 would be expected to bind to one milliliter of the DC Bead LUMI assuming a 1:1 drug to sulfonate group of the bead interaction (Table 2).

**Table 2:** Amount of drug loaded per mL of DC Bead LUMI.

Drug	Doxorubicin HCl*	Gardiquimod	DSR 6434	Imiquimod	BMS-202
Maximum drug loaded, mg drug/ml beads	204.54 ± 3.87	65.97 ± 1.54	65.95 ± 6.96	65.28 ± 3.09	148.05 ± 2.24
mM drug/ml beads equivalent of drug loaded to sulfonated bead	0.35 ± 0.01	0.21 ± 0.00	0.16 ± 0.02	0.27 ± 0.01	0.35 ± 0.01
Theoretical maximum loading level based on SO_3_^−^ contents (mg)	166.5	89.95	114.94	68.97	120.4
Drug: sulfonate	1.228	0.733	0.574	0.946	1.223

*Maximum loading reflects the amount of each drug in its basic form except for Dox which is available in its salt form.

**Figure 3:**
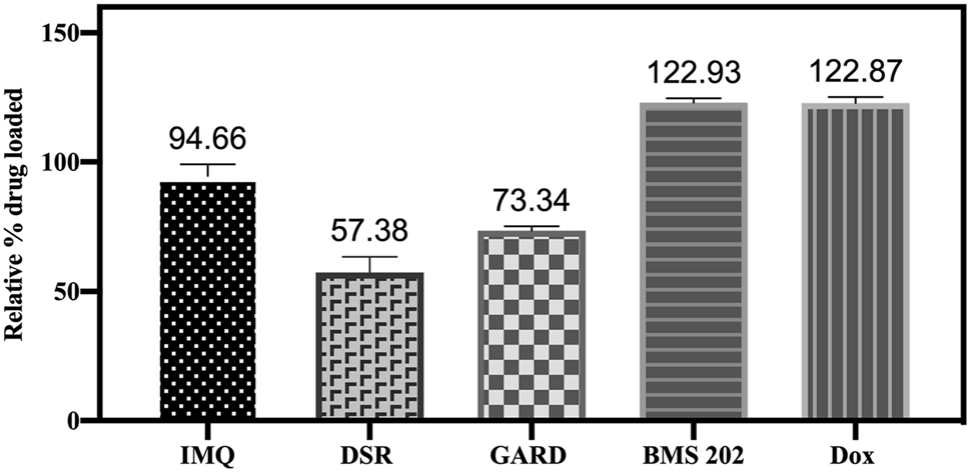
Relative percent drug loaded in DC Bead LUMI. The relative percent drug loaded was defined as the maximum amount of drug loaded as a percent of the amount of SO_3_^−^ ions available in the beads on a molar basis. The results are presented as the mean ± SD (n = 3).

**Figure 4:**
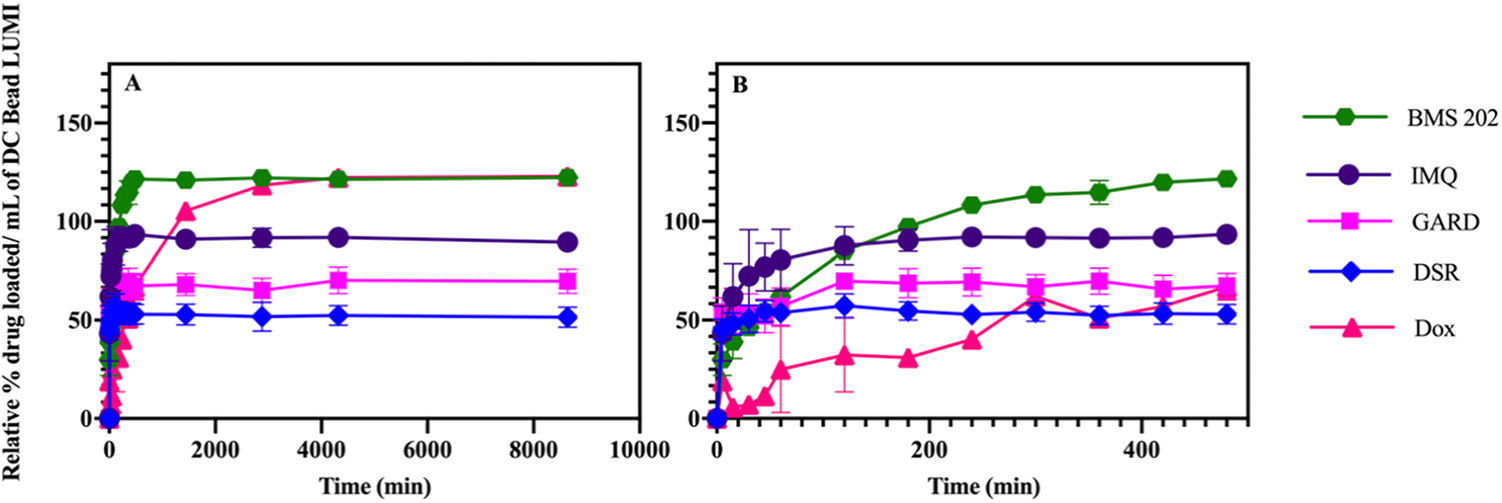
(**A**) Relative percent drug loaded in DC Bead LUMI over time and (**B**) at early time points. The relative percent drug loaded was defined as the maximum amount of drug loaded as a percent of the amount of SO_3_^−^ ions available in the beads on a molar basis. The results are presented as the mean ± SD (n = 3).

**Figure 5:**
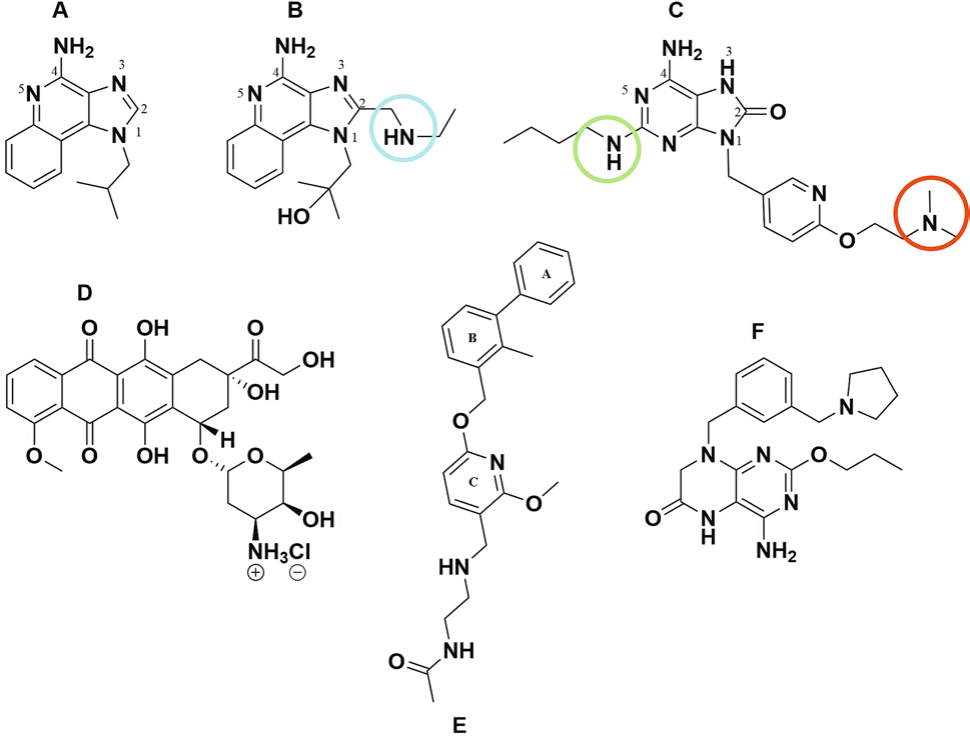
Chemical structures of drugs used in this study: (**A**) IMQ, (**B**) GARD, (**C**) DSR 6434, (**D**) Dox.HCl, (**E**) BMS-202 and (**F**) GS-9620. Circles represent the site of electronic interaction between beads and GARD or DSR 6434.

In this study, drug loading in DC Bead LUMI did not follow a 1:1 drug salt: sulfonate binding. As can be seen in Figs. 3 and 4, Dox and BMS-202 loading resulted in approximately 23% more drug loaded than the predicted capacity of the bead, 122.87 ± 2.32% and 122.97 ± 1.86%, respectively, suggesting that additional mechanisms other than ionic interaction contribute to loading, such as hydrophobic interaction between the hydrophobic drug and the phenyl group on the beads. Loading of Dox was slower than BMS-202. In comparison to BMS-202, Dox has a sugar molecule protruding away from its aglycone moiety^56^ (Fig. 5) introducing steric hinderance which may slow its interaction with the sulfonate groups on the beads. This may explain the slower drug loading with the bead-drug mix requiring an extended period for the drug to access the negatively charged sulfonates (Fig. 4). Similar observation was reported where 0.08 mmol of irinotecan, a spatially slender molecule, was loaded to DC Bead LUMI within 10 min; while it took up to 2–3 h (depending on the bead size) under constant agitation to load 0.065 mmol of Dox per milliliter of beads^42^. A potential explanation may be that the structure of the drug molecule interacting with the bulky triiodo phenyl group introduces steric hinderance which slowed the drug-sulfonate interaction.

The basic difference between LC Bead and DC Bead LUMI is that the latter contains a 2, 3, 5-triiodo phenyl group (Fig. 2) which imparts image-ability, intrinsic hydrophobicity, and steric bulkiness to DC Bead LUMI. These hydrophobic domains, in addition to ionic interactions with bead sulfonate groups, are believed to provide the second force, promoting a process of drug-bead association and stabilization that may drive more drug loading into DC Bead LUMI. Hydrophobic drug-drug interactions may also contribute to drug loading within the beads which may be greater within the dense structure of DC Bead LUMI in which bound drug molecules may be in close proximity to each other^57^. The strength of the hydrophobic force depends on molecular size, shape, and the nature of the chemical bonds and temperature^58–60^. Thus, greater than 1:1 (drug: sulfonate) loading of BMS-202 and Dox may be the result of such hydrophobic or drug-drug interactions. The average amount of Dox loaded into 1 mL of LC Bead was 33.58 ±3.08 mg which was slightly lower than a previously reported amount of 39.0 ± 3.8 mg/mL^55^. One potential cause was the loss of LC Bead due to bead adhesion to the wall of the pipette tips during bead aliquot measurement. This is far less than the maximum loading of Dox in DC Bead LUMI (205 ± 4 mg/mL) likely due in part to its higher density of sulfonate groups, and greater hydrophobic drug-bead and drug-drug interaction effects described above.

In contrast to Dox and BMS-202 loading, maximum occupation of sulfonate groups was just 57% for DSR 6434 and 73% for GARD (Table 2 and Fig. 4) suggesting multiple charges on these drugs may interact with more than one sulfonate group on the beads^45,51^ or steric hinderance may preclude this interaction (Figs. 5). Nitrogen groups of GARD and DSR 6434 (Fig. 5, see colored circles) have substituents that stabilize the cations during acid-assisted dissolution of the drugs which promote ionic interaction with sulfonate groups in the beads. However, these substituents may also hinder the resulting cationic form of the drug from interacting with the sulfonate counter ions on the beads. Whereas occupation of 95% of sulfonate groups by the less hindered IMQ may be due to more favorable drug-bead interaction.

### Bead appearance

**Figure 6:**
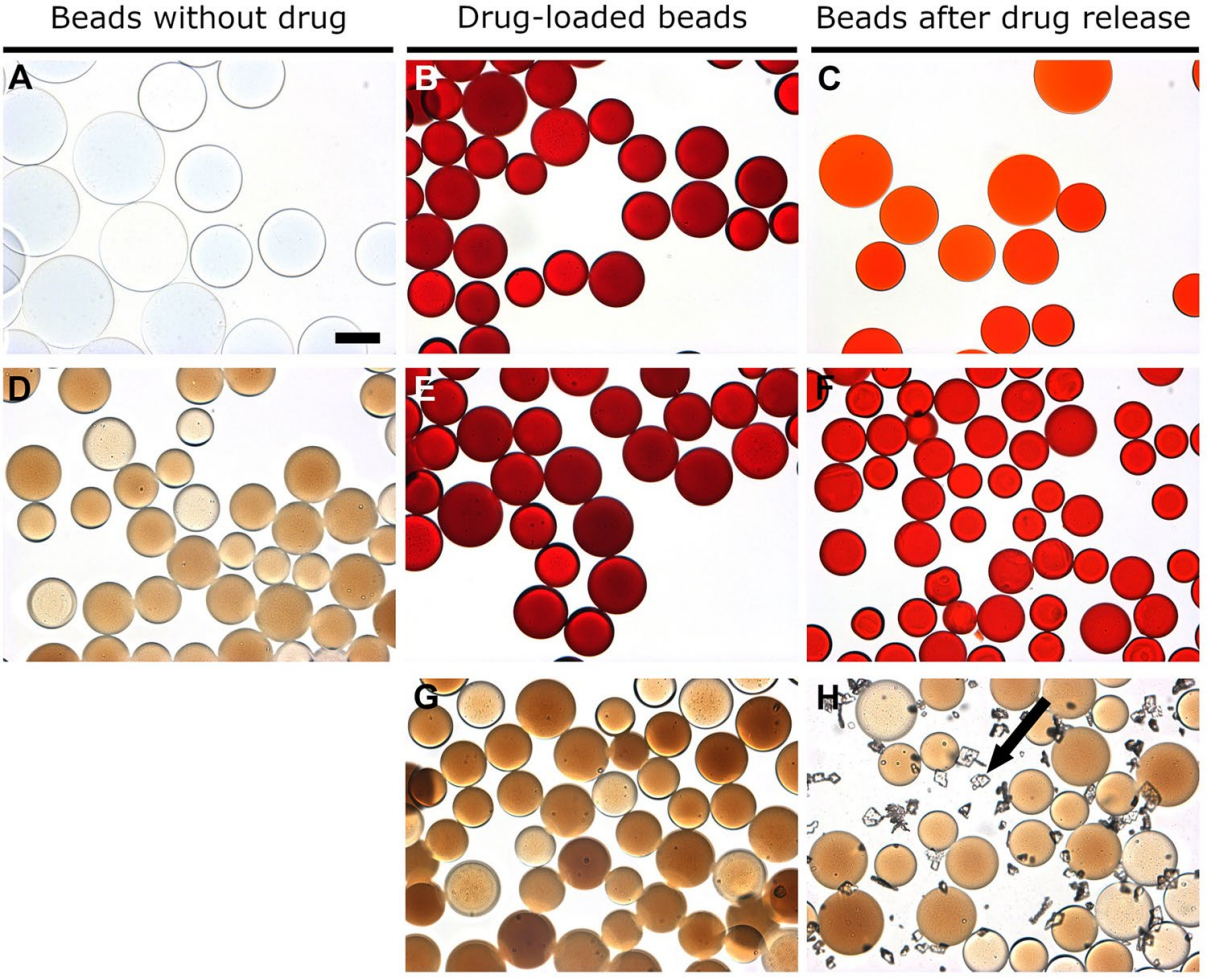
Microscopic images of: (**A**) bland LC Bead, (**B**) Dox-loaded LC Bead, (**C**) LC Bead after Dox release, (**D**) bland DC Bead LUMI, (**E**) Dox-loaded DC Bead LUMI, (**F**) DC Bead LUMI after Dox release, (**G**) IMQ loaded DC Bead LUMI, and (**H**) IMQ-loaded DC Bead LUMI after washing in water and subsequent elution in saline (arrow indicates IMQ aggregates). Scale bar is 100 µm and is the same for all images.

No differences in appearance and color were observed between pre- and post- drug loaded DC Bead LUMI beads for all drug-loaded beads except Dox. Dox-loaded beads were red, since Dox is red, but the TLR agonists and CPI are colorless and thus imparted no color to the resulting drug-loaded beads (Fig. 6). The reddish color of beads in Fig. 6C,F illustrates that a substantial amount of Dox remained in the beads at the end of the elution study.

### In vitro drug elution

**Figure 7:**
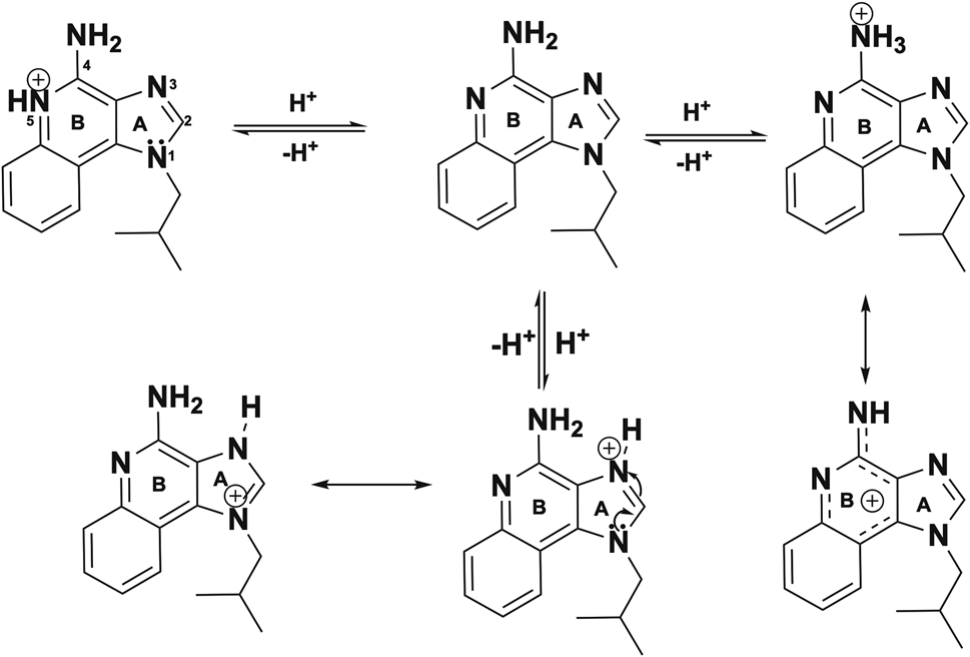
Resonance stabilization of imidazoquinolinium ion and charge localization for the IMQ drug.

**Figure 8:**
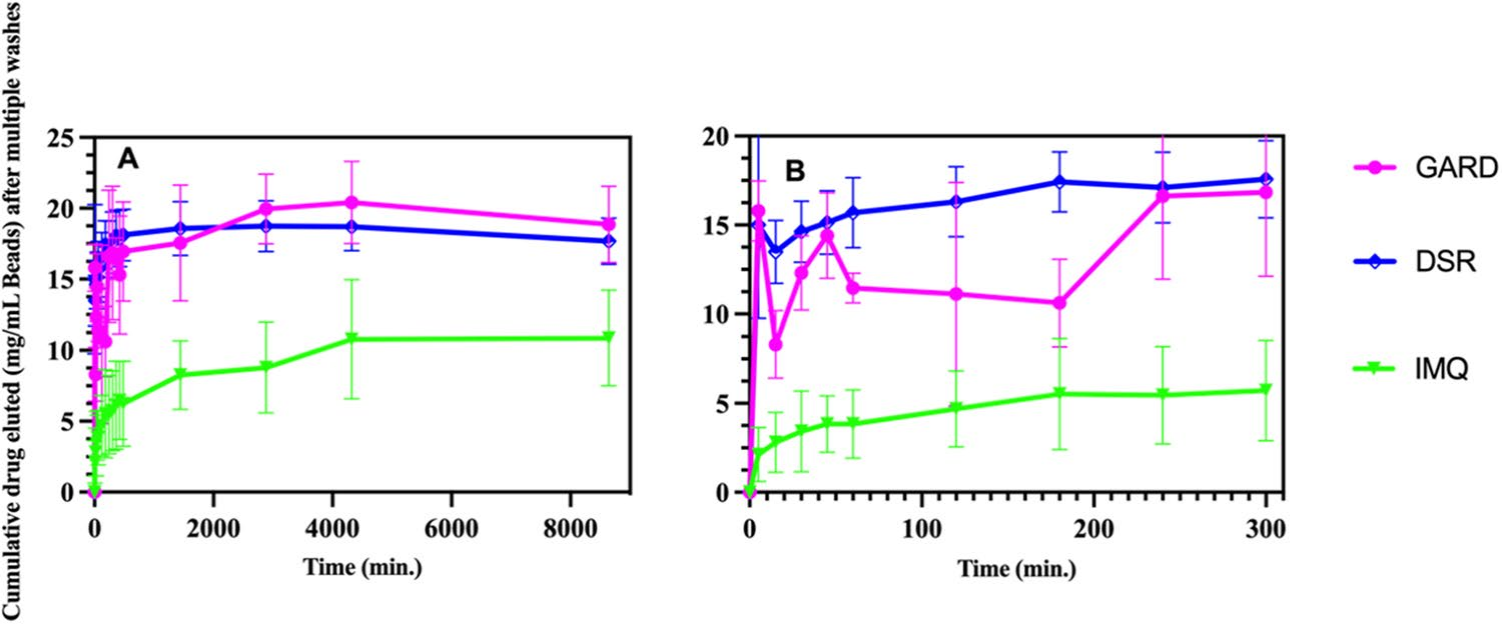
Gardiquimod (GARD) and DSR 6434 (DSR), and imiquimod (IMQ) release in saline from DC Bead LUMI (mg/mL DC Beads LUMI) after multiple washes in deionized water (**A**). Early time points (**B**). The results are presented as the mean ± SD (n = 3).

Elution studies of IMQ, GARD and DSR 6434 from DC Bead LUMI demonstrated that these drugs could be partially removed from the beads by repeated washing with deionized water, suggesting weak binding to the beads. IMQ formed aggregates upon dissociating from the beads during washing (Fig. 6H) which may have affected the released drug quantification. The weak binding of the TLR drugs is explained by resonance and electronic factors. For example, IMQ is an imidazoquinoline fused [4,5-c] carrying isobutyl and amino substituents at N-1 and C-4 respectively (Fig. 5). As indicated in Fig. 7, IMQ has a loan pair of electrons on N-1 which is resonance stabilized into ring A while the charge on the amine at C-4 and nitrogen 5 are delocalized into ring B of the imidazoquinoline, thus its electron pair is not available for forming an ionic bond to a proton during salt formation in acidic solution. As a result, IMQ salt (protonated form) is loosely bound to the sulfonate of DC Bead LUMI which makes the IMQ-bead complex weak so that it rapidly dissociates upon washing with deionized-water. Thus, the amount of TLR drug present in the beads could be variable at the initiation of drug release studies in saline, depending on the number of washes and the amount of drug released during bead preparation for the release study. In contrast, the amount of BMS-202 or Dox present in the bead should be more predictable as the free drug was removed during washing with deionized water with no further drug coming off by continued washing. Figure 8 indicates the release in saline of IMQ, GARD and DSR 6434 that remained after repeated washes in deionized water, suggesting appreciable amount of these drugs had washed out from the bead during the washing process. Quantification of either the washed out or bound IMQ to the beads was difficult, as the drug aggregated either in the washing or elution media as seen in Fig. 6H.

The basic structural difference that affects solubilization as well as binding to DC Bead LUMI between IMQ and GARD is the presence of an alkylated secondary amine substituent at the C-2 position of the imidazoquinoline for GARD molecules (Fig. 5, circled in blue). Whilst the alkylated secondary amine of GARD stabilizes the resulting cations formed during salt formation, it also sterically interferes with binding to the sulfonate of the bead. Similarly, electronic and steric effects may explain the weak binding of DSR 6434 to the beads (see red and green circles in Fig. 5).

**Figure 9:**
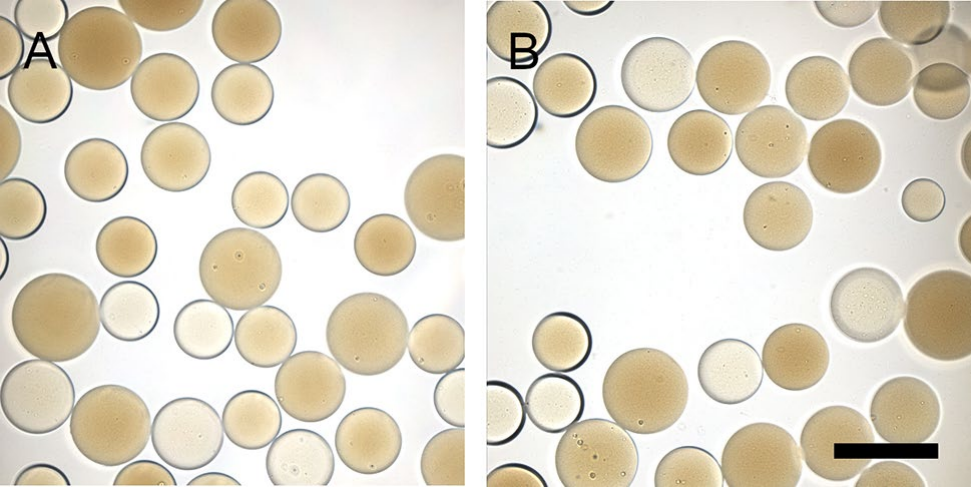
Microscopic images of (**A**) GARD-loaded and (**B**) DSR 6434-loaded DC Bead LUMI after multiple washes in deionized water followed by drug elution in saline. Scale bar is 200 µm and is the same for both images.

The presence of more amine-containing substituents in both GARD and DSR 6434 (Fig. 5, labelled blue, green or red circles) improves the solubility which prevents aggregation of these molecules compared to IMQ which has less substituents. As a result, more of GARD and DSR 6434 are in the aqueous phase during washing or release study. This is evident by the absence of aggregated GARD and DSR 6434 on microscopic images of drug-loaded DC Bead LUMI after washing with de-ionized water and drug release in normal saline (Figs. 8 and 9) compared to IMQ aggregation from IMQ-loaded DC Bead LUMI treated similarly (Fig. 6H).

**Figure 10:**
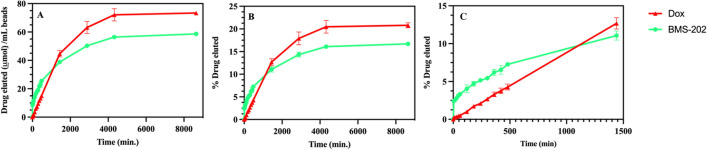
Dox and BMS-202 release profile from DC Bead LUMI over time: (**A**) release in µmole and (**B**) percent drug eluted and (**C**) early time points for (**B**). The results are presented as the mean ± SD (n = 3).

Figure 10 compares the release profiles of Dox and BMS-202 from DC Bead LUMI. In general, the slow release for Dox and BMS-202 compared to the TLR agonists tested may be attributed to strong ionic interaction of Dox and BMS-202 with bead sulfonate groups, hydrophobic interactions between drug molecules and hydrophobic components of the beads, and potential drug-drug interactions within the beads^61–63^. However, the release of BMS-202 (on molar basis) was greater than Dox within the first 5 min (Fig. 10) followed by a similarly slow release. The two reported measures reflect different facets of elution. One is the percentage of drug that can be eluted (% of total loaded) which informs understanding of the bioavailability of loaded drug. The other is the total amount of drug released, which has clinical relevance since that would be the amount of drug delivered per mL of beads.

**Figure 11:**
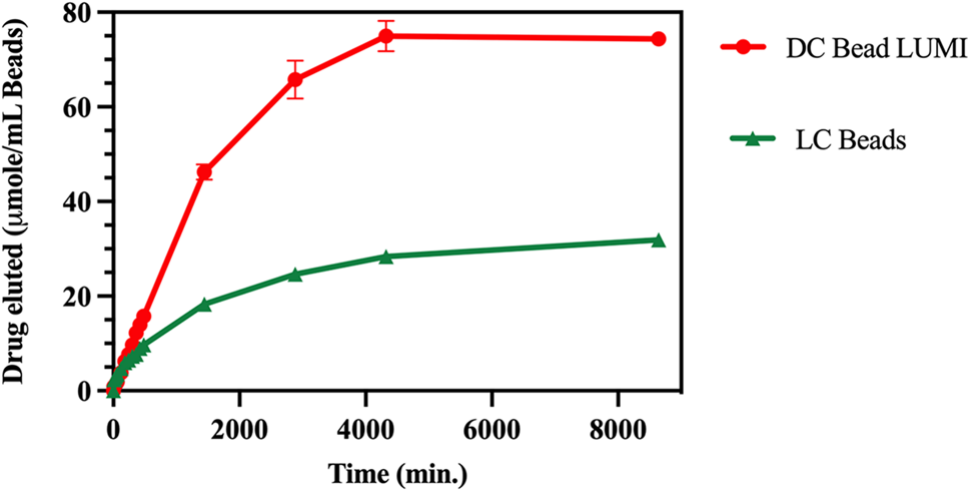
Comparison of Dox released from DC Bead LUMI 74.4 ± 0.5 µmol Dox/ mL DC Bead LUMI) compared to LC Bead (31.9 ±0.3 µmol Dox/ mL LC Bead) over time. The results are presented as the mean ± SD (n = 3).

Although substantial amounts of both drugs were released (Fig. 10A), a majority remained sequestered in the beads after one week in the release media (Fig. 10B). Ashrafi et al., observed a faster release of irinotecan from LC Bead compared to DC Bead LUMI of the same bead size^42^ indicating the likely influence of the hydrophobic and bulky 2,3,5-triiodophenyl group on the DC Bead LUMI on drug release as well as its greater density and reduction in pore size compared to LC Bead. In addition, the presence of a larger amount of sulfonate groups in DC Bead LUMI compared to LC Bead, which may promote greater rebinding of released drug to the bead, may also play a role in retarding the release^62^. The molar amount of drug released from DC Bead LUMI is substantially higher due to its greater loading capacity (Fig. 11) than LC Bead under similar release conditions.

In this study we evaluated the maximum loading capacity of various immune-modulating drugs in radiopaque embolic beads. However, the optimal amount of drug to be loaded into the beads will depend on the desired clinical application, drug release kinetics from the beads, and individual drug pharmacodynamics, and may theoretically be substantially less than the maximum drug loading capacity of the beads. Subsequent preclinical dose escalation and safety studies would be required to estimate safe and effective doses of different drug-bead combinations before clinical use. Regardless, post-release, precipitation or the temporal and spatial dynamics of the immune response and modulation could have major influences upon local and/or systemic therapeutic windows.

## Conclusion

Radiopaque embolic beads (DC Bead LUMI) can be used for loading and release of immune-modulating drugs including TLR agonists and a small molecule CPI. However, the TLR agonists IMQ, GARD, and DSR 6434 demonstrated weak binding to DC Bead LUMI resulting in partial extraction of drug from the beads in water, and rapid release from the beads in saline. IMQ also exhibited aggregation both in deionized water and saline making it potentially unsuitable for this application. GS-9620 was not stable under the conditions used for salt formation, as a result further study was discontinued. The prolonged release profile exhibited by BMS-202 may enable extended localized drug delivery that may serve to reduce peak plasma drug concentrations and increase drug levels within embolized tumors. Maximum drug loading and the amount and rate of release of BMS-202 were comparable with the standard locoregional HCC chemotherapeutic, Dox. Although speculative, effects of steric hindrance and hydrophobicity of drugs and bead may influence the maximum drug loading into and amount released over time from the bead. The combination of local delivery of immune-modulating drugs and drug-eluting microspheres (immuno-beads) may be worthy of further development and translational inquiry towards eventual image-guided immuno-TACE for HCC.

## Supplementary Information

Supplementary Information 1.

Supplementary Information 2.

Supplementary Information 3.

Supplementary Information 4.

## Data Availability

All data generated or analyzed during this study are included in this published article [and raw data are included as supplementary information files]. Additional data, if any, may be shared upon reasonable request from the corresponding author.
