# Author Correction: In vitro characterization of immune modulating drug-eluting immunobeads towards transarterial embolization in cancer

**DOI:** 10.1038/s41598-023-30659-z

**Published:** 2023-03-02

**Authors:** Ayele H. Negussie, Andrew S. Mikhail, Joshua W. Owen, Natalie Hong, Camella J. Carlson, Yiqing Tang, Kendal Paige Carrow, Michal Mauda-Havakuk, Andrew L. Lewis, John W. Karanian, William F. Pritchard, Bradford J. Wood

**Affiliations:** 1 Center for Interventional Oncology, Radiology and Imaging Sciences, NIH Clinical Center, National Institutes of Health, Bethesda, MD USA; 2 Biocompatibles UK Ltd (a BTG International Group Company), Lakeview, Riverside Way, Watchmoor Park, Camberley, GU15 3YL Surrey UK; 3 National Cancer Institute, National Institutes of Health, Bethesda, MD USA

Correction to: *Scientific Reports* https://doi.org/10.1038/s41598-022-26094-1, published online 19 December 2022

The original version of this Article contained an error in the Abstract.

“Maximum drug loading was 204.54 ± 3.87, 65.28 ± 3.09, 65.95 ± 6.96, 65.97 ± 1.54, and 148.05 ± 2.24 mg of drug per milliliter of DC Bead LUMI for Dox, GARD, DSR 6434, IMQ, and BMS-202, respectively.”

now reads:

“Maximum drug loading was 204.54 ± 3.87, 65.97 ± 1.54, 65.95 ± 6.96, 65.28 ± 3.09, and 148.05 ± 2.24 mg of drug per milliliter of DC Bead LUMI for Dox, GARD, DSR 6434, IMQ, and BMS-202, respectively.”

The original version of this Article also contained an error in Table 2, where the “Maximum drug loaded, mg drug/ml beads” value was incorrect for “BMS-202”. The incorrect and correct value appears below.

Incorrect:

**Table:** 

Drug	BMS-202
Maximum drug loaded, mg drug/ml beads	147.19 ± 2.19

Correct:

**Table:** 

Drug	BMS-202
Maximum drug loaded, mg drug/ml beads	148.05 ± 2.24

The original Article has been corrected.

